# Role of DCAF8 in Mammary Ductal Elongation and Branching Morphogenesis

**DOI:** 10.1007/s10911-026-09604-z

**Published:** 2026-03-06

**Authors:** Qianying Han, Miaomiao Ban, Ting Zhang, Tianyun Yu, Xingyue Yan, Yumeng Hu, Pengfei Wu, Yanfang Ma, Luzheng Xu, Yanrong Su, Li Li, Mo Li, Genze Shao

**Affiliations:** 1 Department of Cell Biology, School of Basic Medical Sciences, Peking University Health Science Center, Beijing, 100191 China; 2 State Key Laboratory of Female Fertility Promotion, Center for Reproductive Medicine, Department of Obstetrics and Gynecology, Peking University Third Hospital, Beijing, 100191 China; 3 Department of Gynecology, The First Affiliated Hospital of Zhengzhou University, Zhengzhou, 450000 China; 4 Center of Medical and Health Analysis, Peking University Health Science Center, Beijing, 100191 China; 5 The Irma H. Russo, MD Breast Cancer Research Laboratory, Fox Chase Cancer Center-Temple University Health System, Philadelphia, PA 19111 USA; 6 Department of Breast and Thyroid Surgery, Southwest Hospital, Third Military Medical University (Army Medical University), Chongqing, 400038 China

**Keywords:** Dcaf8, Mammary gland, Progesterone receptor, Morphogenesis, CRL4 E3 ligase, Knockout

## Abstract

Postnatal mammary gland development involves the formation of a highly branched epithelial ductal tree, primarily through the elongation and branching morphogenesis of mammary epithelial ducts. Multiple factors are involved in this process, in which both estrogen and progesterone receptors (ER/PR) play a crucial role. In this study, we identified a role of DCAF8 in promoting mammary ductal elongation and branching through its impacts on ER/PR signaling. Homozygous *Dcaf8* knockout mice exhibited significant delay in mammary ductal elongation during puberty, which was characterized by a reduction in mammary ductal elongation area and distance, and terminal end buds (TEBs); abnormal branching morphogenesis of mammary ducts was also observed in adult *Dcaf8* null mice, which was characterized by a reduction of lateral branches and an increase of ductal bifurcation. To further elucidate the mechanism underlying DCAF8’s role in mouse mammary development, we performed transcriptomic sequencing and biochemical experiments. The results revealed that downstream key effectors of the PR signaling pathway were significantly downregulated, while the expression of ERβ, a potential inhibitor of ERα/PR signaling, was significantly elevated in the mammary gland of *Dcaf8* null mice. Collectively, this study suggests that DCAF8 may play an important role in mammary development by promoting ductal elongation and branching morphogenesis, through ERβ-mediated inhibition of ERα/PR signaling pathway.

**Supplementary Information:**

The online version contains supplementary material available at 10.1007/s10911-026-09604-z.

## Introduction

The mammary gland exhibits remarkable plasticity, with majority of its development occurring postnatally. It undergoes extensive expansion during puberty and then cyclic remodeling through estrous cycles pregnancy, lactation, and involution [1]. It is a branched epithelial exocrine organ where mammary epithelial ducts are composed of luminal epithelial cells or alveolar cells arranged on the inside, surrounded by an outer layer of basal cells (or myoepithelial cells) [2]. At birth, the primary mammary ductal tree occupies only a small portion of the entire fat pad, remaining relatively quiescent until puberty [3]. Postnatally, under the influence of ovarian hormones and growth hormones, the mammary ducts of pubertal mice rapidly develop, forming terminal end buds (TEBs) at the duct ends, which mediate ductal elongation and bifurcation. In adult, mammary glands mainly form tertiary branching ducts through side branching. Adult mammary epithelial ductal tree fills the entire fat pad, and the TEBs structures disappear [2, 4].

During puberty, the formation of TEBs and elongation of mammary ducts are primarily regulated by estrogen signaling and growth hormone [5]. The estrogen receptor (ER) is a critical transcription factor that regulates epithelial cell proliferation and ductal morphogenesis during postnatal mammary gland development. There are two forms of intracellular estrogen receptors, α and β, in which α is the major receptor that regulates ductal morphogenesis [6]. Amphiregulin (AREG) and transcription factors like GATA3 and FOXA1 have been demonstrated to play crucial roles in the formation of TEBs and in promoting ductal growth [7–11]. Additionally, during puberty, progesterone signaling enhances TEBs expansion, ductal elongation, and lateral branching through the modulation of WNT4 or in synergy with the insulin-like growth factor 1 receptor (IGF1R). In adulthood, the morphogenesis of lateral branches is mainly controlled by progesterone and progesterone receptor signals, with key effectors including the receptor activator of nuclear factor κB ligand (RANKL), cyclin D1 (CCND1), and WNT4, while transcription factors such as Inhibitor of DNA binding protein 2 (ID2) and Forkhead box O1 (FOXO1) also play an important role [12–15]. The regulation of mammary development is multifaceted, and the mechanisms governing this process are largely unclear.

Ubiquitination is a reversible post-translational modification that plays a role in various biological processes including mammary development and tumorigenesis [16–18]. Many E3 ubiquitin ligases have been found to regulate mammary development, and mutation or loss of their function is associated with breast cancer. For example, deletion of Brca1 from mouse mammary epithelial cells resulted in blunted ductal morphogenesis and tumor formation [18]; F-box and WD repeat domain containing 7 (Fbxw7) deletion in mammary epithelial cells resulted in severe atrophy of mammary glands and a lactation defect, and triggered development of mammary tumor [19]; Cullin 3 (Cul3) deficient mouse mammary gland exhibited delayed ductal growth and abnormal epithelial structure and dilated terminal end buds [18]; X-linked inhibitor of apoptosis protein (XIAP)-deficiency leads to delayed lobuloalveolar development [20]. The mechanism underlying how these E3 ligases regulate mammary gland development is largely unclear. One possibility is that these E3 ligase may target key regulators or transcription factors involved in the development of mammary gland. For example, BRCA1 was reported to ubiquinate estrogen receptor α (ERα), a critical transcription factor governing mammary gland development [21], while FBXW7 controls proteasome-mediated degradation of Notch signaling, which plays essential role in breast development and tumorigenesis [22].

DCAF8 (DDB1 and Cul4-associated factor 8), also known as WDR42A (WD repeat-containing protein 42 A), is a specific substrate receptor for CRL4 E3 ubiquitin ligases [23]. The functions of DCAF8 have been implicated in several crucial biological processes including reproduction, aging, neuropathies, tumorigenesis, and chemotherapy resistance [24–29]. In the liver, CRL4-DCAF8 ubiquitinates histone H3K79 and promotes methylation modifications at H3K9, driving epigenetic silencing of fetal liver genes [27]. The DCAF8 p.R317C mutation is identified as a genetic cause of the rare giant axonal neuropathy of axonal hereditary motor and sensory neuropathy (HMSN2), potentially related to the degradation of neurofilaments [30]. DCAF8 also plays a critical role in spermatogenesis, *Dcaf8 knockout* mice exhibit significant morphological abnormalities and typical bent-head deformities. Sperm movement characteristics, including progressive motility, path velocity, progressive velocity, and track speed, were significantly lower in *Dcaf8 knockout* mice than in wild-type (WT) mice [24]. As the E3 ubiquitin ligase for the chromatin remodeling factor LSH, CRL4-DCAF8 participates in the epigenetic regulation of ferroptosis [25]. While DCAF8 is involved in multiple pathophysiological processes, the role of DCAF8 in mammary development remains underexplored.

Recently we identified a negative role of DCAF8 in regulating ERβ through ubiquitination [29]. Since ERβ was demonstrated to play a role in suppressing the activity of ERα [31], which is critical for mammary ducts extension and lobuloalveolar differentiation, this result suggests that DCAF8 may have important functions in the development of mammary gland. Moreover, two paralogues of human DCAF8, DCAF8L1 and DCAF8L2, were found to negatively regulate the protein stability of BRCA1 and/or BARD1 [32, 33], two proteins that are involved in mammary development and breast cancer [17, 34]. These data together suggest that DCAF8 may have important functions in mammary gland development. Here we employed *Dcaf8* systemic knockout and mammary epithelial conditional knockout and knock-in mice to investigate the role of DCAF8 in mammary development.

## Results

### Expression Patterns of DCAF8 and Generation of *Dcaf8*-Modified Mice

To investigate the biological function of DCAF8, we analyzed the expression patterns of DCAF8. We utilized publicly available transcriptome sequencing data from 27 normal human tissues (BioProject: PRJEB4337 [35]) and found that members of the DCAFs family are widely present in human tissues, but their expression patterns vary. DCAF8, in comparison to other DCAFs, exhibited higher expression levels in more than half of the 27 human tissues (Fig. S1), indicating that DCAF8 is a broadly expressed substrate receptor of CRL4. We then assessed the distribution of DCAF8 in various mouse tissues using immunohistochemistry. The results revealed that DCAF8 is widely distributed across mouse tissues and organs (Fig. S2A-H), with high expression levels in mouse ovarian granulosa cells and mammary ductal luminal epithelial cells (Fig. S2B&D). These findings suggest that DCAF8 may have important biological functions, especially in the female reproductive system.

**Fig. 1:**
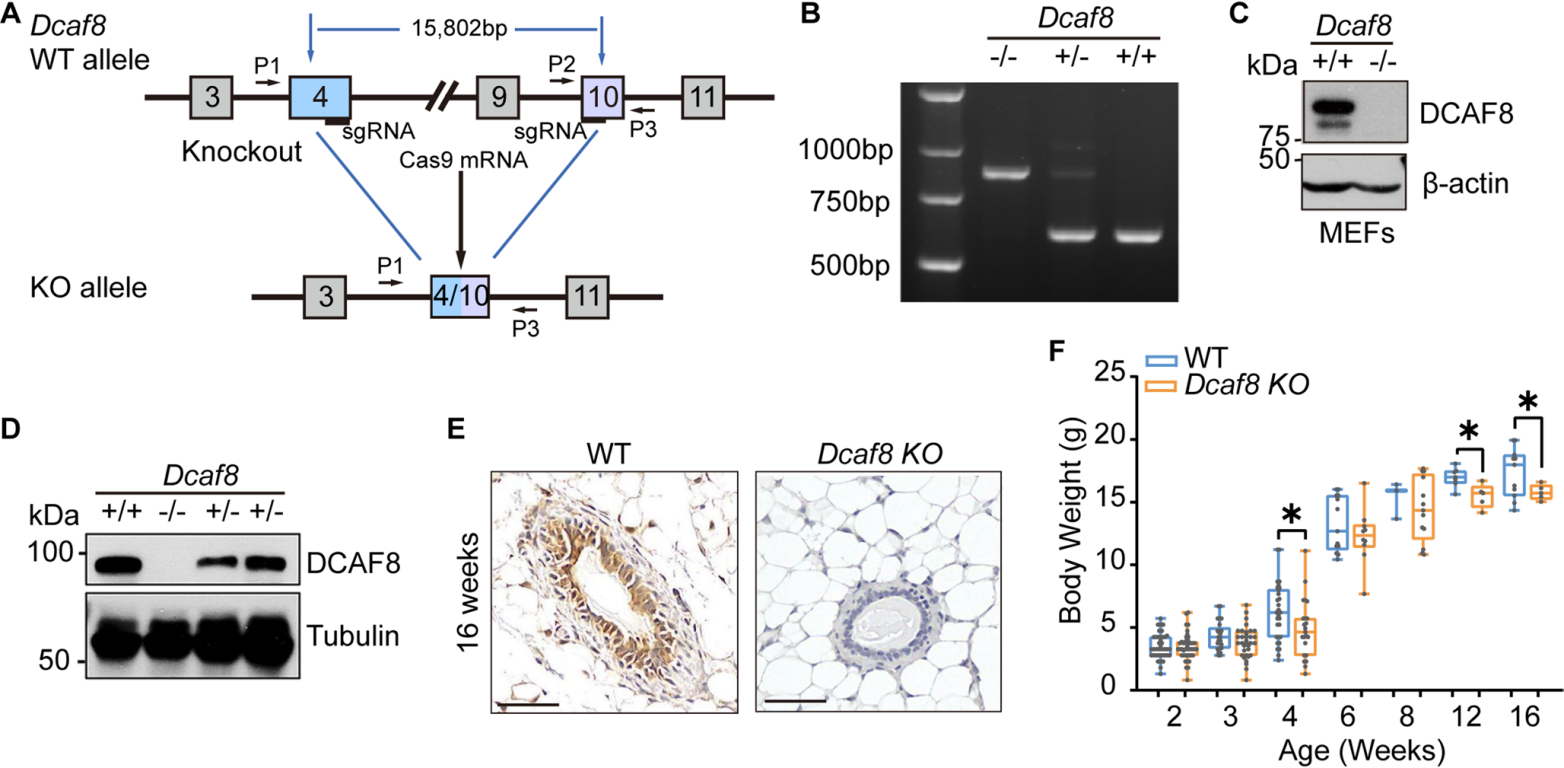
Generation and Confirmation of *Dcaf8 KO* mouse model. **A** Schematic diagram of the construction of *Dcaf8 KO* mouse. **B** *Dcaf8* knockout mice genotyping. Mouse genomic DNA were used for genotyping by PCR. The PCR product size for WT was 619 bp (P2/P3), and for *Dcaf8 KO*, 893 bp (P1/P3). **C** Confirmation of *Dcaf8 KO* by Western blotting (WB). The expression of DCAF8 in mouse embryonic fibroblasts (MEFs) from *Dcaf8 KO* or WT mice was analyzed by WB. **D** Western blotting analysis of DCAF8 expression in mammary gland tissues from *Dcaf8 KO*, heterozygous and WT mice. **E** Confirmation of *Dcaf8 KO* by immunohistochemistry staining of mammary gland with DCAF8 antibody. **F** Growth rate of *Dcaf8 KO* mice. The body weight of WT and *Dcaf8 KO* mice (including both males and females) at different ages was recorded and box-plotted. Boxes: 25th and 75th percentiles, whiskers: minimum and maximum, central lines: medians; statistical analysis was performed using two-tailed *t*-tests. *P < 0.05. Scale bar, 50 μm (**E**); WB, western blotting; WT, wild type; IHC, immunohistochemistry

To investigate the functions of DCAF8 in vivo, we generated *Dcaf8* systemic knockout (*KO*) mouse, in which the genomic DNA between the 3’ end of exon 4 and 5’ end of exon 10 of the *Dcaf8* gene was deleted using sgRNA/Cas9 technology (Fig. 1A &B). *Dcaf8 KO* mice (*Dcaf8*^−/−^) were obtained by cross-breeding of heterozygous mice (*Dcaf8*^+/−^). Western blotting analysis showed that DCAF8 was absent in mouse embryonic fibroblast cells (MEFs) and mammary gland tissues from these *Dcaf8 KO* mice (Fig. 1C&D). Loss of DCAF8 was further confirmed by immunohistochemistry (IHC) staining of mammary glands of *Dcaf8 KO* mice (Fig. 1E). DCAF8 is mainly expressed in epithelial cells in wild-type (WT) mammary gland, but completely lost in *Dcaf8 KO* mice. Notably, a similar expression pattern of DDB1 and Cul4A, two components of CRL4, were also detected in mammary epithelial cells (Fig. S3). Genotypic distribution of neonatal litters in heterozygous *Dcaf8*^+/−^ mouse breeding conformed to Mendel’s law, suggesting no premature lethality of knockout mice. However, some of *Dcaf8 KO* pups died within three weeks after birth, while others survived. Male *Dcaf8 KO* mice were infertile, which is consistent with previous report [24]. In addition, *Dcaf8 KO* mice generally had lower body weights at each different developmental stage when compared to WT mice (Fig. 1F), suggestive of an important role of DCAF8 in mouse development.

### *Dcaf8 KO* Female Mice Exhibit Delayed Mammary Ductal Elongation During Puberty

The mouse mammary gland undergoes many phases of development and differentiation during its lifetime. This process occurs mainly during puberty, when the ductal epithelium expands to form an organized mammary tree [36]. The terminal end buds (TEBs) at this stage bifurcate repeatedly and invade into surrounding fatty stroma, and mature ducts sprout laterally to form side branches [37]. Therefore, TEBs bifurcation, ductal elongation and side-branching are three major events in mammary gland development. Mouse mammary gland development usually attains maturity at age of 8–12 weeks [38].

**Fig. 2:**
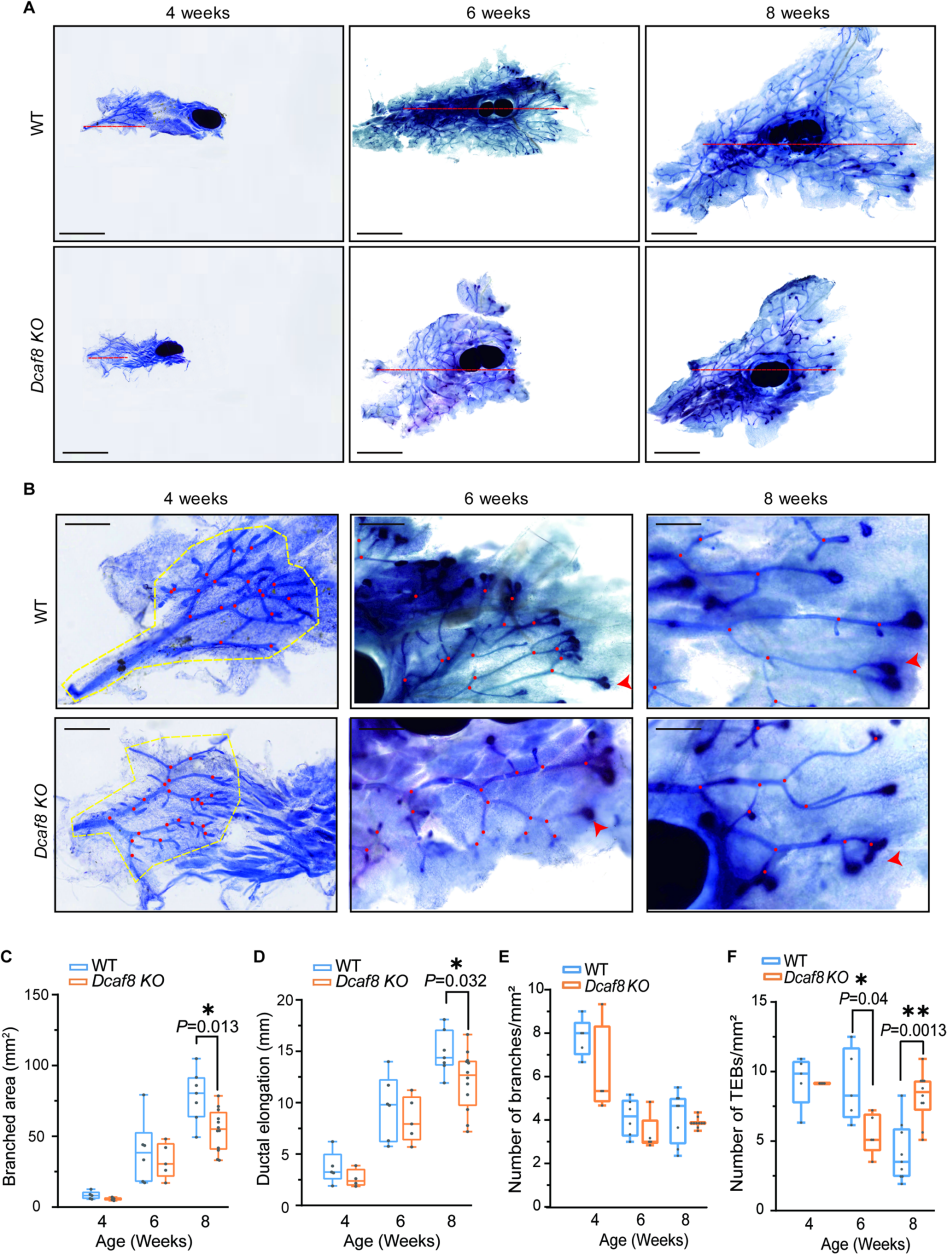
DCAF8 regulates mouse mammary gland morphogenesis during puberty. **A** Whole mount toluidine blue staining of the fourth mammary glands from WT and *Dcaf8 KO* mice at 4 (WT: *n* = 5; *Dcaf8 KO*: *n* = 4), 6 (WT: *n* = 6; *Dcaf8 KO*: *n* = 5), and 8 (WT: *n* = 7; *Dcaf8 KO*: *n* = 12) weeks old. Red lines indicate the distances of mammary ductal extension. **B** Magnified view of the local structures in the mammary gland from panel **A**. Yellow dashed circles highlight the overall area of ductal extension. Red dots mark the branching points of the mammary ducts. Red arrowheads indicate typical terminal end buds (TEBs) structures. **C**-**F** Quantitative analysis of the whole mount staining results. Mammary ductal tree branched area (**C**) was measured using ImageJ. The overall extension area of the mammary ducts (mm^2^) in each of the panel **B** was circled in yellow dashed lines. Mammary ductal elongation distance (**D**) was measured from the origin of the duct to the farthest edge, as indicated by the red lines in panel **A**. The number of mammary duct branches (**E**) and TEBs structures (**F**) in the mammary ducts were determined as the average from three individual fields of view (FOV) per gland; Boxes: 25th and 75th percentiles; whiskers: minimum and maximum; central lines: medians. Statistical analysis was performed using two-tailed *t*-tests. **P < 0.01, *P < 0.05; Scale bar: 2.5 mm (**A**), 1 mm (**B**); WT, wild type

To explore the role of DCAF8 in mouse mammary development, we first examined the effects of *Dcaf8 KO* on mammary duct development during puberty. The fourth inguinal mammary glands were obtained from pubertal WT and *Dcaf8 KO* mice, and mammary whole-mount staining was performed (Fig. 2A&B). A quantitative analysis of mammary development was conducted at various developmental stages, including 4, 6 and 8 weeks old. The result showed that mammary development in *Dcaf8 KO* mice was notably delayed throughout puberty when compared to WT mice. *Dcaf8 KO* mice exhibited reduced mammary ductal branched area and ductal elongation distance, especially at 8 weeks old (Fig. 2C&D).

The formation of the pubertal mammary ductal tree requires not only rapid proliferation and expansion of epithelial cells, but also branching morphogenesis, especially the bifurcation occurring at the terminal end buds (TEBs) [39]. The ductal branches were fewer in *Dcaf8 KO* mice during puberty compared to wild-type mice (Fig. 2E). At 4 and 6 weeks old, the number of TEBs in *Dcaf8 KO* mouse mammary glands was generally fewer than that in WT mice; however, at 8 weeks old, when the TEBs gradually regressed in WT mice, the number of TEBs in *Dcaf8 KO* mice did not decrease but instead increased (Fig. 2F). These results indicate that mammary development is a dynamic process, and the developmental retardation observed in *Dcaf8 KO* mice mammary development may be gradually recovered over time as mice matured (Fig. S4).

In summary, the absence of DCAF8 leads to delayed mammary development in pubertal mice, significantly with decreased branched area and elongation distance of the mammary ductal tree, decreased branching, and delayed TEBs development.

### *Dcaf8* Conditional Knockout and Knock-In Mice Exhibit Abnormal Mammary Development

**Fig. 3:**
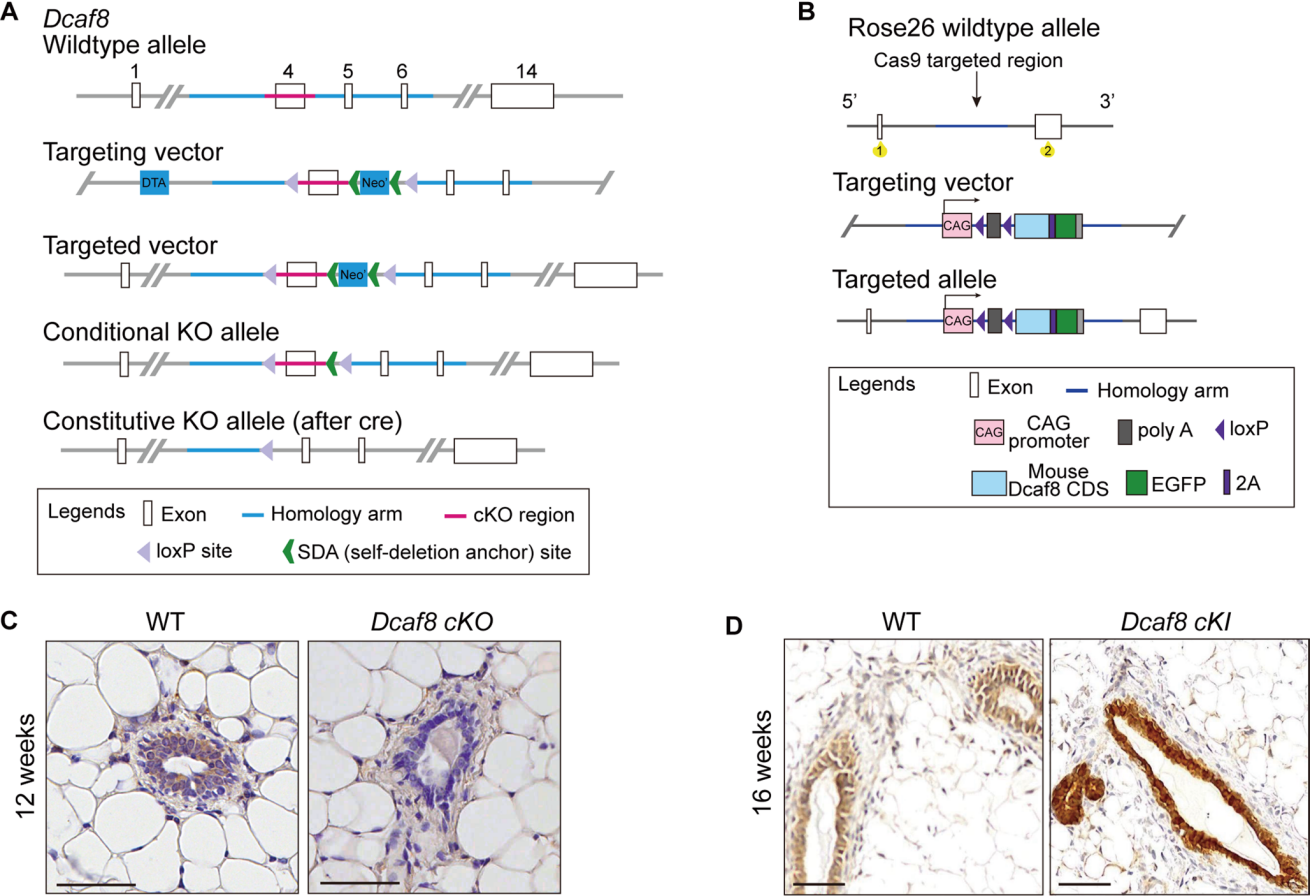
Generation and confirmation of *Dcaf8* conditional Knock-in and Knock-out mice. **A** Schematic diagram of the generation of *Dcaf8* conditional knock-out mice. **B** Schematic diagram of *Dcaf8* conditional knock-in at the locus of ROSA26 in C57BL/6 mice. **C** IHC staining of DCAF8 in the mammary gland tissues from WT and *Dcaf8* conditional knock-out mouse. **D** IHC staining of DCAF8 in the mammary gland tissues from WT and *Dcaf8* conditional knock-in mouse. Scale bar, 50 μm (**C**-**D**); WT, wild type; IHC, immunohistochemistry

The morphogenesis of postnatal mammary duct is driven by both luminal and basal lineage of progenitors that are derived from the embryonic mammary stem cells [40]. These mammary stem/progenitor cells express Keratin 5 or 14 (K5/14), and Keratin 14 promoter driven-Cre has been successfully used for the expression or ablation of target genes in many mice models [40–42]. DCAF8 is mainly expressed in the luminal epithelial cells of mammary ducts (see Fig. 1E). To further determine the role of DCAF8 in mammary development, particularly its function within mammary epithelial cells, we first generated *Dcaf8*-floxed mice (*Dcaf8* ^*loxP/loxP*^) in which two loxP sites spanning the exon 4 of *Dcaf8* were introduced (Fig. 3A). Conditional deletion of DCAF8 was achieved in the mammary epithelial cells of *Dcaf8* conditional knockout mice (*cKO*) by crossing *Keratin14-Cre (K14-Cre)* with *Dcaf8*-floxed mice. Similarly, we generated *Dcaf8* knock-in mouse model (*Dcaf8 KI*) in which a CAG-loxP-mouse *Dcaf8* CDS-EGFP cassette was introduced in the first intron of *Rosa26* gene on chromosome 1 (Fig. 3B). DCAF8 over-expression was obtained in the mammalian epithelial cells of *Dcaf8 cKI* mice by crossing *Dcaf8 KI* mice with *K14-Cre* mice. Mouse genotyping was shown in Fig. S5A-B. *Dcaf8 cKO* and *Dcaf8 cKI* mice were confirmed by IHC and Immunofluorescence staining analysis of DCAF8 expression in the mammary gland tissues (Fig. 3C-D & Fig. S5C).

**Fig. 4:**
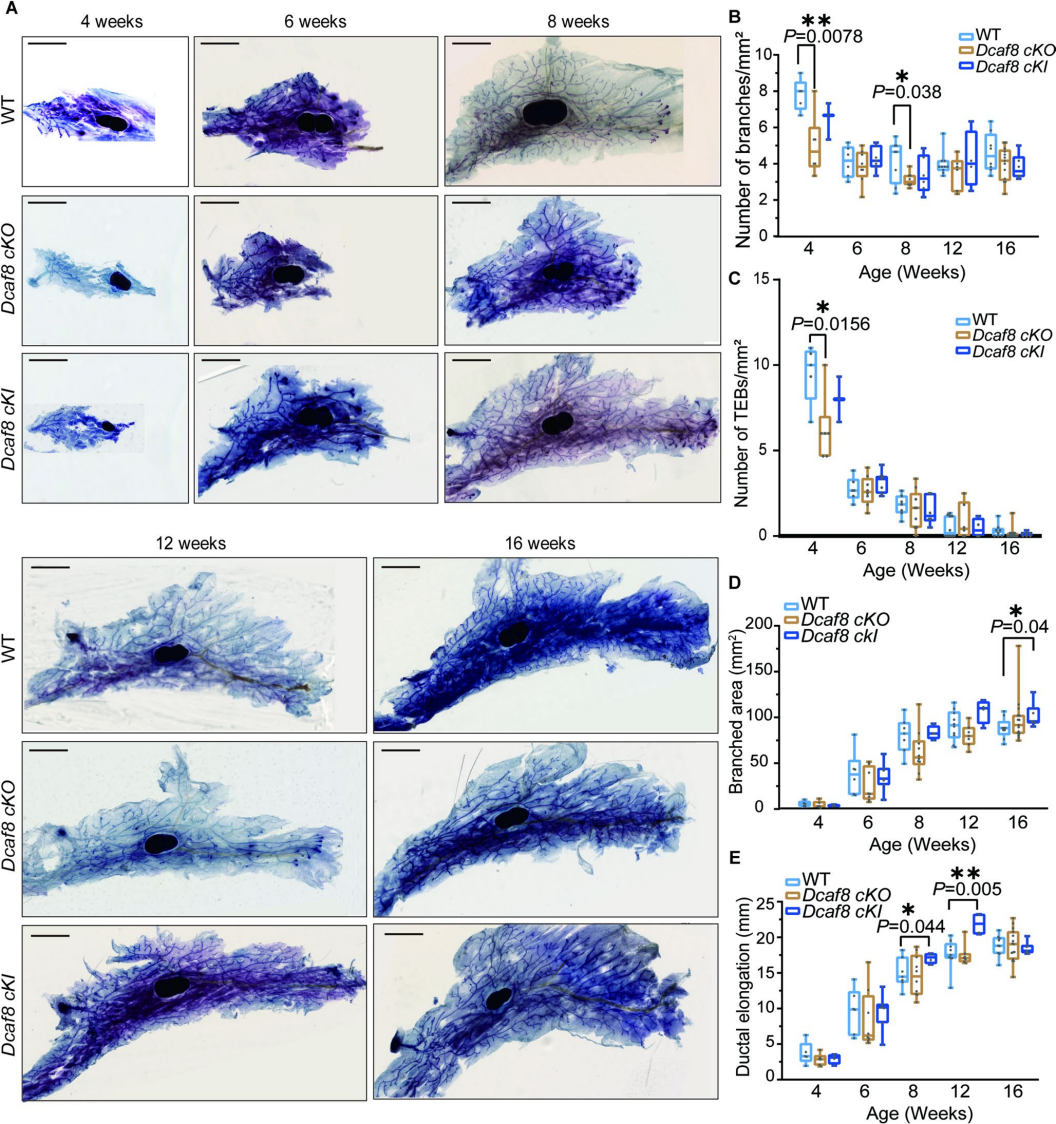
Abnormal mammary gland development phenotypes in *Dcaf8* conditional Knock-in and Knock-out mice. **A** Whole mount toluidine blue staining of the fourth mammary glands from WT, *Dcaf8 cKO* (conditional Knock-out), and *Dcaf8 cKI* (conditional Knock-in) mice at various developmental stages (For 4 weeks old: WT, *n* = 5; *Dcaf8 cKO*, *n* = 6; *Dcaf8 cKI*, *n* = 4. For 6 weeks old: WT, *n* = 6; *Dcaf8 cKO*, *n* = 8; *Dcaf8 cKI*, *n* = 7. For 8 weeks old, WT, *n* = 7; *Dcaf8 cKO*, *n* = 10; *Dcaf8 cKI*, *n* = 4. For 12 weeks old, WT, *n* = 10; *Dcaf8 cKO*, *n* = 7; *Dcaf8 cKI*, *n* = 4. For 16 weeks old, WT, *n* = 9; *Dcaf8 cKO*, *n* = 14; *Dcaf8 cKI*, *n* = 7). **B**-**E** Quantitative analysis of the whole mount staining results of the mammary glands as shown in panel **A**. The number of mammary duct branches (**B**) and TEBs structures (**C**) in the mammary ducts were determined as the average from three individual fields of view (FOV) per gland. **D** Mammary ductal tree branched area was quantified using ImageJ, the overall extension area of the mammary ducts (mm^2^) was selected. **E** Mammary ductal elongation distance was measured from the origin of the duct to the farthest edge (mm). Boxes: 25th and 75th percentiles; whiskers: minimum and maximum; central lines: medians; statistical analysis was performed using two-tailed *t*-tests. **P < 0.01; *P < 0.05. Scale bar, 2.5 mm (**A**). WT, wild type

A detailed analysis of mammary development of these mice was conducted. *Dcaf8 cKO* mice exhibited some delayed mammary duct development phenotypes observed as in *Dcaf8 KO*, including decreased total number of ductal branches at 4 & 8 weeks, compared to WT mice (Fig. 4A&B). Significant decreased TEB numbers were observed in *Dcaf8 cKO* mice at 4 weeks old, which were recovered thereafter (Fig. 4A&C). Branched area and ductal elongation in *cKO* mice were slightly decreased with no significance (Fig. 4D&E). These results suggest that conditional knockout of *Dcaf8* in mammary gland partially recapitulated the duct development delay phenotypes observed in *KO*. In contrast, overexpression of DCAF8 in the epithelial cells significantly promote mammary gland development. *Dcaf8 cKI* mice exhibited rapid mammary development with significant longer ductal elongation distances at 8 & 12 weeks old when compared to WT (Fig. 4E). These results indicate that overexpression of DCAF8 in mammary epithelial cells promotes the elongation and expansion of mammary ducts.

### Ductal Branching is Abnormal in Adult *Dcaf8 KO* Female Mice

During the development of mouse mammary gland, new branches sprout laterally from the primary ducts, a process known as lateral or side branching [43]. In adulthood, the mammary ducts of mice primarily form short tertiary branches through lateral branching under the stimulation of progesterone [44]. To further investigate whether the effects of DCAF8 deletion on pubertal mammary development persist into adulthood and whether DCAF8 influences adult mammary development, particularly lateral branching, we analyzed mammary development of virgin WT and *Dcaf8 KO* female mice at 16 weeks old. As expected, mammary glands of wild type mice developed normally and completely formed a mature mammary gland tree at 12 and 16 weeks (Fig. S4A&B). TEBs that were observed in *Dcaf8 KO* mice at 8 weeks of age, were almost completely disappeared at 16 weeks old (Fig. S4C), suggesting the pubertal developmental delays observed in *Dcaf8 KO* mice (8 weeks old) were almost completely recovered in adulthood (12 and 16 weeks old) (Fig. S4D-F). Moreover, compared with WT mice, *Dcaf8 KO* mice displayed fewer lateral branches, and more bifurcations, suggesting that DCAF8 might promote TEBs growth, duct elongation and side-branching. This process might be compromised when DCAF8 was lost.

**Fig. 5:**
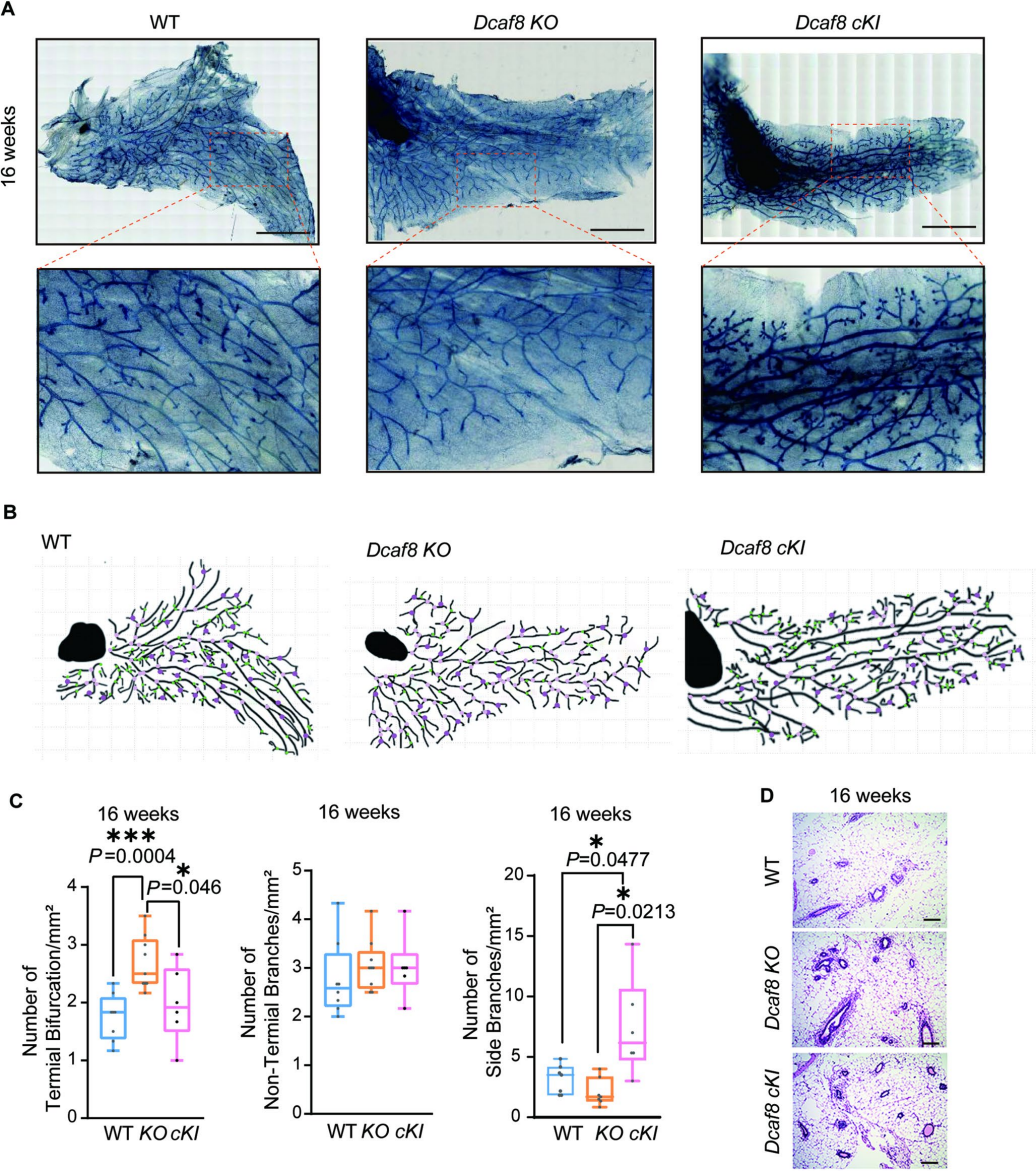
DCAF8 is required for mammary gland branching morphogenesis in adult mice. **A** Whole mount toluidine blue staining of the mammary glands from WT, *Dcaf8 KO* and *cKI* mice at 16 weeks of age. The lower panel shows a magnified view. **B** Schematic diagraph of mammary gland ductal trees of 16-week-old female mice. Potential sites of ductal side branching were marked as green dot. Potential non-terminal branching points were marked as pink dot. Assumptive terminal bifurcation points were marked as purple dot. **C** Statistical analysis of the number of terminal bifurcation (left panel) and non-terminal branching points (middle panel) or side branches (right panel) in the mammary ducts. For bifurcation analysis and non-terminal branches point analysis, WT (*n* = 8), *Dcaf8 KO* (*n* = 9) and *cKI* (*n* = 6) mice at 16 weeks of age were used; for side branches number analysis, WT(*n* = 7), *Dcaf8 KO* (*n* = 7) and *cKI* (*n* = 6) mice at 16 weeks of age were used. The numbers of bifurcation or side branches were determined as the average number of three individual fields of view (FOV) per gland. statistical analysis was performed using two-tailed *t*-tests. *P < 0.05. **D** Hematoxylin and eosin (H&E) staining of mammary gland tissues showing the structure of mammary gland of WT and *Dcaf8 KO* mice at 16-week-old. Scale bars, 2.5 mm (**A**); 100 μm (**D**)

We also observed an increased terminal bifurcation in *Dcaf8 KO* mice. Potential bifurcation sites, including sites on the TEBs that in the middle of bifurcation and sites just freshly bifurcated (terminal bifurcation), non-terminal branching points, and lateral branching (small branches that extend from the sides of ducts) were counted and compared. Significant increase in the number of terminal bifurcation sites was found in *Dcaf8*^*−/−*^ mice. No significance in the number of non-terminal branching points was found. Concomitantly lateral branching was markedly inhibited in these mice (Fig. 5A-C). The duct morphology was further analyzed by Hematoxylin and eosin (H&E) staining of mouse mammary gland tissues (Fig. 5D). In contrast to the phenotype observed in *Dcaf8 KO* mice, *Dcaf8 cKI* mice exhibited significant increase of side branches when compared with WT and *KO* mice, suggesting that overexpression of DCAF8 in mammary epithelial cells promotes duct side branching. Taken together, these results suggest that DCAF8 is required for mammary glands branching morphogenesis in adult mice.

### Progesterone Receptor Signaling Pathway is Downregulated in *Dcaf8 KO* Mice

**Fig. 6:**
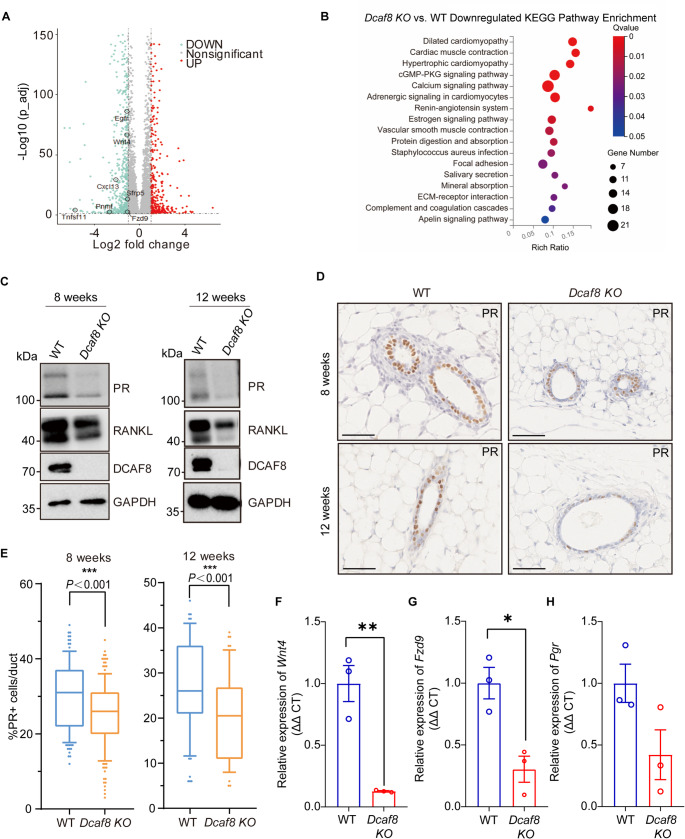
Significant downregulation of key effectors in the Progesterone Receptor pathway in *Dcaf8 KO* mouse mammary glands. **A** Transcriptome sequencing of mammary glands from 8-week-old WT and *Dcaf8 KO* mice, with a volcano plot displaying differentially expressed genes. Green dots represent significant downregulated genes, red dots represent significant upregulated genes, and gray dots indicate genes with no significant changes. Key effectors of the progesterone receptor signaling pathway are highlighted with black circles. **B** KEGG pathway enrichment analysis of downregulated genes in *Dcaf8 KO* versus WT mammary tissue. **C** WB analysis of mammary gland tissues from 8-week-old and 12-week-old WT and *Dcaf8 KO* mice. The indicated antibodies were used. **D**&**E** Proportion of PR positive cells in the mammary ducts. IHC staining of PR was performed in mammary gland tissues (**D**). PR positive cells were counted and its percentage of the total mammary epithelial cells in each duct was plotted for 8-week-old and 12-week-old WT and *Dcaf8 KO* mice (**E**). Data are presented as mean ± standard deviation. Statistical analysis was performed using two-tailed *t*-tests. ***P < 0.001. **F**-**H** Analysis of mRNA levels of *Wnt4* (**F**), *Fzd9* (**G**), and *Pgr* (**H**) in mammary gland from 8-week-old WT and *Dcaf8 KO* mice. Data are presented as mean ± standard deviation. Statistical analysis was performed using two-tailed *t*-tests. **P < 0.01; *P < 0.05. Scale bar, 50 μm (**D**). WT, wild type

To address the molecular mechanisms by which DCAF8 regulates mouse mammary development and its downstream effector molecules, we extracted RNA from mammary tissues of 8 weeks old virgin female WT and *Dcaf8 KO* mice for RNA sequencing analysis. Genes including *Egfr*, *Wnt4*, *Tnfsf11*, *Fzd9*, *Sfrp5*, *Cxcl13*, *Pnmt*, and *Mybpc1* were differentially downregulated in *Dcaf8 KO* mouse mammary glands (Fig. 6A). Since these genes are key effectors of the progesterone receptor signaling pathway [45], we therefore believe that PR signaling may have been affected by DCAF8 knockout. In addition, KEGG analysis also identified the enrichment of ERα signaling pathway (Fig. 6B &Fig. S6). Based on that PR is a downstream target gene of ERα signaling pathway, these results suggesting loss of DCAF8 may influence ERα signaling pathway.

RANKL and WNT4 are the key paracrine effectors downstream of the progesterone receptor, and are involved in progesterone receptor-mediated processes such as mammary ductal side branching and TEBs formation. They also regulate mammary epithelial cell stemness, proliferation, and ductal differentiation [12, 46]. Consistently, reduced expression levels of the progesterone receptor and RANKL signals were seen in *Dcaf8 KO* mouse mammary (Fig. 6C). *Dcaf8 KO* mice exhibited significantly lower levels of PR expression in the mammary ducts during both puberty and adulthood, with a significantly decrease in the proportion of PR-positive cells (Fig. 6D&E). In addition, RT-qPCR showed that the transcription levels of *Wnt4* and homologous ligand *Fzd9* were significantly reduced in the *Dcaf8 KO* mammary (Fig. 6F-H*)*. These results suggest that PR-mediated RANKL and WNT4 signaling pathways is downregulated in *Dcaf8 KO* mice and may play an important role in DCAF8-mediated mammary development.

### Downregulation of PR Signaling is Associated with an Aberrant Expression of ER in *Dcaf8 KO* Mice

**Fig. 7:**
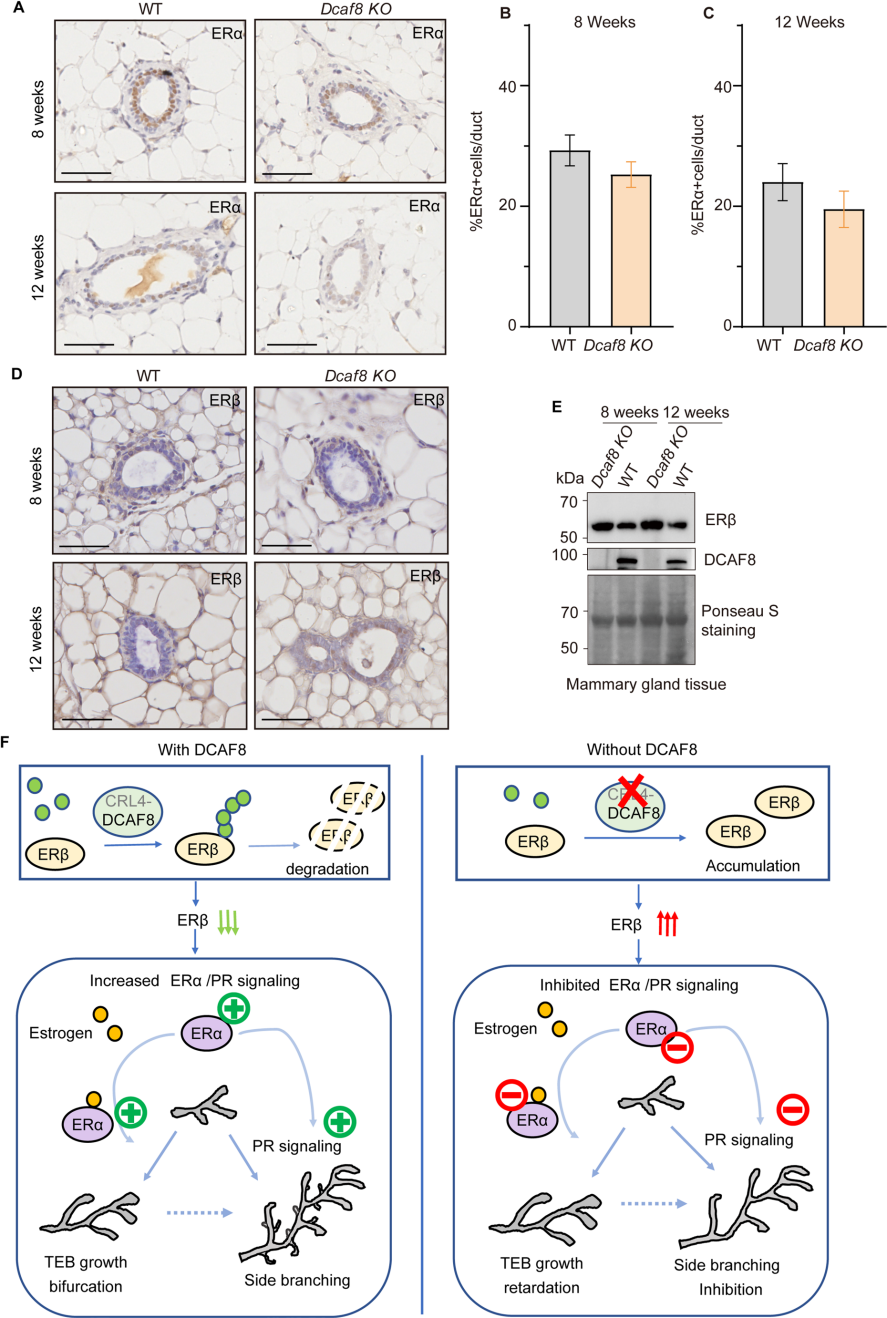
Aberrant ER expression in the mammary glands of *Dcaf8 KO* mice. **A** Representative images of IHC staining of ERα of mammary gland tissues from 8-week-old and 12-week-old WT and *Dcaf8 KO* mice. **B**-**C** Statistical analysis of the proportion of ER positive cells in the mammary ducts. The percentage of ER positive cells in the total mammary epithelial cells in each duct for 8-week-old (**B**) and 12-week-old WT (**C**) and *Dcaf8 KO* mice was calculated and plotted. Data are presented as mean ± standard deviation. Statistical analysis was performed using two-tailed *t*-tests. **D** IHC of ERβ was performed with the mammary gland tissues from WT and *Dcaf8 KO* mice. Representative images of IHC staining of ERβ in mammary gland tissues from 8-week-old and 12-week-old WT and *Dcaf8 KO* mice. **E** WB analysis of ERβ expression in the mammary gland tissues from WT and *Dcaf8 KO* mice. **F** The schematic model illustrates the role of DCAF8 in the morphogenesis of mammary glands. In mammary gland development, ER/PR signaling is essential for mammary duct TEBs growth and ductal elongation, and duct side branching. These signaling are limited by ERβ, which can form heterodimers with ERα and inhibit its activity and downstream PR signaling. DCAF8 may play a role in the regulation of mammary gland development through negative regulation of ERβ stability, the level of which is important in antagonizing ERα/PR signaling. In the absence of DCAF8 as is exemplified in *Dcaf8 KO* mice, the development of terminal end buds (TEBs) during puberty is delayed, ductal elongation and expansion are inhibited. In addition, the branching morphogenesis is also abnormal in adult *Dcaf8 KO* mice, with a significant reduction in lateral branching, leading to a mammary ductal tree primarily composed of bifurcations. Scale bar, 50 μm (**A**&**D**)

During postnatal mammary gland development, both estrogen and progesterone are key regulators of mammary gland development, promoting the growth and branching of mammary gland ducts by binding to their receptors [47]. Progesterone receptor is an ERα-induced gene [48]. To investigate whether the downregulation of PR signaling is associated with ER signaling, we assess the expression of estrogen receptors (ER) and its relation with progesterone receptors (PR) in the mammary ducts of both wild-type and *Dcaf8 KO* mice. Like the low expression of PR, ERα expression levels were also slightly reduced in the mammary glands of *Dcaf8 KO* mice, accompanied by a decrease in ERα-positive cells (Fig. 7A-C). In contrast to ERα, the expression of ERβ was significantly increased in *Dcaf8 KO* mice (Fig. 7D&E), which is consistent with our previous report showing ERβ as a substrate for CRL4-DCAF8 [29]. Since ERβ was reported to play a negative role in regulation of ER signaling by antagonizing ERα, these results suggest that both the reduction of ERα and the increased ERβ due to lack of DCAF8 may synergistically inhibit PR expression and its downstream signaling in the mammary ductal epithelium, thus contributing to the abnormal mammary development observed in *Dcaf8 KO* mice.

## Discussion

By using *Dcaf8*-modified mouse models we demonstrated that DCAF8 participates mammary gland development through ERβ-mediated inhibition of PR signaling. *Dcaf8* knockout mice exhibit delayed mammary ductal elongation during puberty and reduced lateral branching in adulthood, and simultaneously decreased proportion of PR positive cells in these mammary ducts, suggesting that DCAF8 may execute its function in the development of mammary gland through regulating PR signaling (Fig. 7F). Consistent with our findings, previous study has shown that loss of PR function impaired mammary development. AF1_FFF mice, in which the AF1 domain of *Pgr* was mutated and lack of PR activity, exhibited significantly reduced mammary TEBs, decreased lateral branches, and shortened ductal length [49]. These phenotypes in mammary gland development were like that of *Dcaf8 KO* mice in this study. These findings suggest that the function of DCAF8 in mammary gland development is associated with its impacts on progesterone signaling pathway. Indeed, we found that key effectors of the PR signaling pathway, including *Tnfsf11*, *Wnt4*, and *Fzd9*, were significantly downregulated in mouse breast tissues in the absence of DCAF8. RANKL and WNT4 are the two critical factors critical for mammary gland development. Previous studies have proved that RANKL-mediated paracrine signaling promotes the proliferation of mammary epithelial cells, facilitating the morphogenesis of lateral branches [14, 49, 50], while WNT4 plays a crucial role in the maintenance of stemness in progesterone-mediated mammary stem cells and in the formation of TEBs structures and the morphogenesis of lateral branches by binding to its homologous receptor [12, 51, 52]. The downregulation of RANKL and WNT4 in *Dcaf8 KO* mice reinforces the hypothesis that activation of progesterone receptor signaling pathway is one mechanism for DCAF8-mediated promotion of mammary gland development.

How DCAF8 regulates PR signaling? To explore the underlying mechanism, we first asked whether PR is a substrate of CRL4-DCAF8, one of the E3 ubiquitin ligases that participate in various biological processes through ubiquitinating substrate proteins [25, 27, 53]. However, no direct interaction between DCAF8 and PR was found. We then checked whether ER is affected by DCAF8, because PR is a target gene of ER signaling. As expected, we identified ERβ, but not ERα, as a major gene that differentially expressed in *Dcaf8 KO* mice. This finding is consistent with our previous finding showing that ERβ is a substrate for CRL4-DCAF8 [29]. ERβ is a negative regulator of estrogen signaling, through forming heterodimers with ERα in target cells, ERβ antagonizes ERα-mediated transcription [54–58]. Because *Pgr* is a target gene of ER in both normal mammary epithelial and breast cancer cells [59], we therefore believe that DCAF8-mediated downregulation of ERβ may play a role in antagonizing ERα, which in turn exerts negative impact on the transcription of PR (Fig. 7F). Furthermore, ERβ is crucial for ovarian function. ERβ knockout mice exhibit abnormal follicle development and fail to ovulate [60]. As ovary is an organ to produce estrogens, DCAF8-mediated degradation of ERβ may therefore affect ovarian development and function, and hence the development of mammary gland. Notably, in this study we observed a smaller litter size in *Dcaf8 KO* female mice than in WT (4.5 vs. 8 pups/litter), and the pups of *Dcaf8 KO* female mice usually exhibited a 30% reduction in survival rate relative to WT (71% vs. 100%) in the first three weeks after born. Moreover, male *Dcaf8 KO* mice are infertile. It is plausible that these phenotypes may associate with defective ER/PR signaling due to lack of DCAF8. Further study will be needed to explore this possibility.

Taken together, we demonstrated that DCAF8 plays a role in the development of mammary gland probably through ERβ-mediated inhibition of PR signaling pathway. These findings reveal a biological function of DCAF8 and provide a theoretical basis for our understanding of the molecular mechanisms of mammary development. Since mammary development is closely associated with breast cancer, this study will have implications for breast cancer tumorigenesis and treatment.

## Materials and Methods

### Antibodies

For Western blotting, the following antibodies were used: anti-DCAF8 (#ab176809, abcam; 1:4000 dilution), anti-GAPDH (#5174, 1:10000 dilution), anti-ERβ (#sc-53494, Santa Cruz; 1:1000 dilution), anti-RANKL (#sc-52950, Santa Cruz, 1:1000 dilution), anti-β-actin (#A1978, Sigma-Aldrich, 1:8000 dilution), anti-ERα (#8644S, Cell Signaling Technology, 1:1000 dilution), anti-PR (#8757S, Cell Signaling Technology, 1:1000 dilution). For Imunochemistry and/or Immunofluorescence staining, the following antibodies were used: anti-DCAF8 (Sigma, #HPA027218), anti-ERβ (ThermoFisher, #MA5–24807), anti-ERα (Santa Cruz, #sc-8005), anti-PR (Cell Signaling Technology, #8757S).

### Mice and Genotyping

All animal experiments were performed under the approval of the Peking University Animal Care and Use Committee. *Dcaf8* systemic knockout mice (*Dcaf8 KO*) were created by CRISPR/Cas9-mediated deletion of the DNA spanning exon 4–10 of *Dcaf8* gene in C57BL/6 mice (Mouse-Saving Biosciences, Beijing, China). Heterozygous mice (*Dcaf8*^*+/−*^) were confirmed by PCR and DNA sequencing, and *Dcaf8*^*−/−*^ (*Dcaf8 KO*) mice were obtained through breeding of these heterozygous mice. Conditional *Dcaf8* knockout mice were produced by Cyagen, Shanghai, China. *Dcaf8* floxed mice (*Dcaf8*^*flox/flox*^) in which two LoxP sites flanking the exon 4 of *Dcaf8* gene were crossed with *K14-Cre* mice (provided by Zhengquan Yu lab in China Agricultural University) to generate conditional *Dcaf8* heterozygous mice (*K14-Cre*; *ckoDcaf8*^*flox/−*^), which were further used to generate conditional *Dcaf8* knockout mice, *K14-Cre*; *Dcaf8*^*flox/flox*^ (*Dcaf8 cKO*) mice. *Dcaf8* konck-in mice (Rosa26-*Dcaf8*, EGFP knock-in mice, *Dcaf8* c*KI*) were also produced by Cyagen, Shanghai, China. For the *Dcaf8 cKI* model, the “CAG-loxP-Stop-loxP-mouse *Dcaf8* CDS-2A-EGFP-polyA” cassette was cloned into intron 1 of *Rosa26* gene on chromosome 1 of C57BL/6 mice by CRISPR/Cas9-mediated genome engineering. Conditional over-expression of *Dcaf8* in mammary gland was achieved through crossing *Dcaf8 KI* mice with keratin 14-Cre (*K14-Cre)* mice to generate conditional *Dcaf8* knock-in mice (*K14-Cre; Dcaf8 KI*, *Dcaf8 cKI*).

Genotyping was determined via PCR. The PCR conditions were as follows: 94°C for 5 minutes, followed by 30 cycles of 94°C for 30 seconds, 60°C for 30 seconds, and 72°C for 30 seconds, with a final extension at 72°C for 5 minutes. PCR products were separated using 1.5% agarose gels. For *Dcaf8 KO* mice genotyping, a PCR product of 893 bp for *Dcaf8 KO* allele vs 619 bp for WT allele was amplified. For *Dcaf8 cKI* genotyping, a PCR product of 512 bp for *Dcaf8 KI* allele vs 758 bp for WT allele was amplified. For *Dcaf8 cKO* genotyping, a PCR product of 352 bp for *Dcaf8 cKO* allele vs 238 bp for WT allele was amplified. The following primers were used: For *Dcaf8* knock-out: P1 (*Dcaf8 KO*-F(5’-TTCCACTTAGAAGTTCCCCCAG-3’)), P3 (*Dcaf8*-R (5’-CACGGGAAACAGGCCAGATA-3’)) and P2 (*Dcaf8*-WT-F (5’-GGGTACCTAGAAACCGCATCCCAGACC-3’)); for *Dcaf8* knock-in: Rosa26-WT-F (5’-CAGACTTGTGGGATACAGAAGAC-3’); Rosa26-Mutation-F (5’-AGTCCACCTCACTCCTCATAAC-3’) and Rosa26-R (5’-CTGCTGTCCATTCCTTATTCCATAG-3’). For *Dcaf8 cKO: Dcaf8* cKO-F (5’- AGTATAAGACAGTAGGAGGGAGCA-3’), and *Dcaf8 cKO*-R (5’- ATGGGCTTAGCTTCCTTTGTTTTC-3’). For K14-Cre: *Cre*-F (5’-GCGGTCTGGCAGTAAAAACTATC-3’) and *Cre*-R (5’- GTGAAACAGCATTGCTGTCACTT-3’).

### Whole Mount Toluidine Blue Staining of Mouse Mammary Glands and Quantification

The mouse mammary glands were harvested and fixed in fresh 4% paraformaldehyde for 24–48 h. After treated with acetone for 3–4 days, the glands were sequentially placed in 100%, 95%, and 70% ethanol for 2 h, and then placed in distilled water overnight. Next, the mammary glands were stained with toluidine blue buffer for 3.5 h and then treated with 100% and 70% ethanol for 45 min. After washed with distilled water, the mammary glands were treated with 4% ammonium molybdate for 1 h and then washed with distilled water overnight. Next, the tissues were placed in 70% and 95% ethanol for 2 h and then treated with 100% ethanol twice and each time for 1 h. Last, the tissues were placed in Histoclear (Cat. No. R30148, Yuanye Biotechnology) overnight and this step should not be done on a shaker. The glands were spread out on micro slide and were mounted with neutral gum. Panoramic scanning was performed using a pathology slide scanner for subsequent observation and quantitative analysis. Ductal extension distances and areas were measured using ImageJ. The number of terminal end buds (TEBs) and ductal branches were counted, and calculated as the average from three measurements from three individual fields of view (FOV) each whole mount. Potential bifurcation sites were counted as the sites on the TEBs that in the middle of bifurcation or just freshly bifurcated (terminal bifurcation). Non-terminal branching points and side branches were counted. Side branches were counted as small branches that extend from the sides of ducts, where two asymmetric bifurcated branches emanating from (we define the maximum length of a side branch is 500 μm). The number of bifurcation and side branches were calculated as the average from three measurements from three individual fields of view (FOV) each whole mount.

### Immunohistochemistry Staining

Fresh mouse tissues were fixed in 10% neutral buffered formalin overnight, dehydrated, and embedded in paraffin to prepare 5 μm sections. The tissue sections were deparaffinized in xylene and rehydrated through a graded alcohol series, followed by antigen retrieval in a citrate buffer (pH 6.0) using a microwave. The process involved bringing the solution to a rapid boil for 2–5 min and continuing microwave heating for 10 min (microwave cycling every 20–30 s, with the solution boiling for 10 s each cycle). The sections were allowed to cool for 20–30 min, then washed three times with PBS for 3 min each. The tissues were treated with 3% H_2_O_2_ in the dark for 20 min. They were then covered completely with 10% goat serum and incubated at room temperature for 30 min. After blocking, the sections were incubated with primary antibodies at 4 °C overnight. The primary antibodies used were anti-DCAF8 (Sigma), anti-PR (CST), and anti-ERα (Santa Cruz). The sections were then incubated with biotinylated secondary antibodies and streptavidin-HRP at room temperature for 30 min. After washing with PBS, the tissues were detected by DAB solution, and the reaction was stopped immediately with distilled water. The sections were counterstained with hematoxylin for 3 min, treated with 1% hydrochloric acid in alcohol and 1% ammonia water for 3 min respectively. The slides were then dehydrated through graded alcohol series, followed by xylene, and mounted with a coverslip. The slides were then scanned for observation.

### Western Blotting

Fresh tissues were lysed using RIPA lysis buffer (50 mmol/L Tris-HCl, pH 7.4; 150 mmol/L NaCl; 1% Triton X-100; 1% sodium deoxycholate; 0.1% SDS), with 2% PMSF and protease inhibitors added prior to use. After grinding at -20 °C, the samples were lysed on ice for 20 min. The protein samples were then subjected to SDS-PAGE, and the separated proteins were transferred to a polyvinylidene fluoride (PVDF) membrane. We blocked the membrane with 5% non-fat milk at room temperature for one hour, then incubated it overnight with specific primary antibodies at 4 °C. The membrane was incubated with labeled secondary antibodies at room temperature for one hour. Finally, proteins were detected using an ECL-Plus detection kit. The primary antibodies used were anti-DCAF8 (Abcam), anti-β-actin (Sigma), anti-PR (CST), anti-RANKL (Santa Cruz), anti-Tubulin (Santa Cruz).

### RNA Extraction, RT-qPCR, and RNA-Sequencing

RNA was extracted and converted to cDNA using a Reverse Transcription Kit (MF011-T, mei5bio). Quantitative PCR (qPCR) was performed on an Applied Biosystems 7500 Real-Time PCR System using SYBR Green qPCR Mix (Selleck, B21703). Whole transcriptome sequencing was performed at the Beijing Genome Institute (BGI). The following pairs of primers are used for qPCR analysis: For Dcaf8, 5’AGAGCTCCTGGCCAGTTACA3’ (forward), 5’GGGGCCATAGAAATTGACGC3’ (Reverse); For Fzd9, 5’GCCTGTGTTTTCCAGACCCT3’ (forward), 5’CCCAGA GCGCCCGATTATC3’ (Reverse); For Wnt4, 5’CACGTCTTTACCTCGCAGGA 3’ (forward), 5’GTCAGGATGCTCGGACAACAT3’ (Reverse); For Pgr, 5’GCGAGTA GAATGACAGCTCCTT3’ (forward), 5’GGGGTGGAGGTCGTACAAG3’ (reverse); For Gapdh, 5’GGAAGGTGAAGGTCGGAGTC3’ (forward), 5’ATGAAGGGGTCA TTG ATGGCA3’ (reverse).

### Statistical Analysis

All data were analyzed using the GraphPad prism 8 software. All the experiments were repeated at least three times. For each experiment, we used 3–12 animals. We performed two-tailed unpaired Student’s *t*-tests to compare the data and considered *P* < 0.05 as statistically significant.

## Supplementary Information

Supplementary Material 1.

Supplementary Material 2.

## Data Availability

Data will be made available on request.
