# Supplementary material for: Role of DCAF8 in Mammary Ductal Elongation and Branching Morphogenesis
Source: J Mammary Gland Biol Neoplasia. 2026 Mar 6;31(1):11. doi: 10.1007/s10911-026-09604-z (PMC13065545; doi:10.1007/s10911-026-09604-z)
Supplement: Supplementary file 2 — Supplementary Material 2. [file 10911_2026_9604_MOESM2_ESM.docx]

**Supplementary Figures**

**Role of DCAF8 in mammary ductal elongation and branching morphogenesis**

Qianying Han^1,6#^, Miaomiao Ban^2#^, Ting Zhang^3#^, Tianyun Yu^1^, Xingyue Yan^1^, Yumeng Hu^1^, Pengfei Wu^1^, Yanfang Ma^1^, Luzheng Xu^4^, Yanrong Su^5^, Li Li^1^, Mo Li^2^*, Genze Shao^1^*

^1^Department of Cell Biology, School of Basic Medical Sciences, Peking University Health Science Center, Beijing, 100191, China.

^2^State Key Laboratory of Female Fertility Promotion, Center for Reproductive Medicine, Department of Obstetrics and Gynecology, Peking University Third Hospital, Beijing, 100191, China.

^3^Department of Gynecology, the First Affiliated Hospital of Zhengzhou University, Zhengzhou, 450000, China.

^4^Center of Medical and Health Analysis, Peking University Health Science Center, Beijing, 100191, China.

^5^The Irma H. Russo, MD Breast Cancer Research Laboratory, Fox Chase Cancer Center-Temple University Health System, Philadelphia, PA, 19111, USA.

^6^Department of Breast and Thyroid Surgery, Southwest Hospital, Third Military Medical University (Army Medical University), Chongqing 400038, China.

^#^These authors contribute equally.

*Correspondence whom should be addressed ([gzshao@bjmu.edu.cn](mailto:gzshao@bjmu.edu.cn))


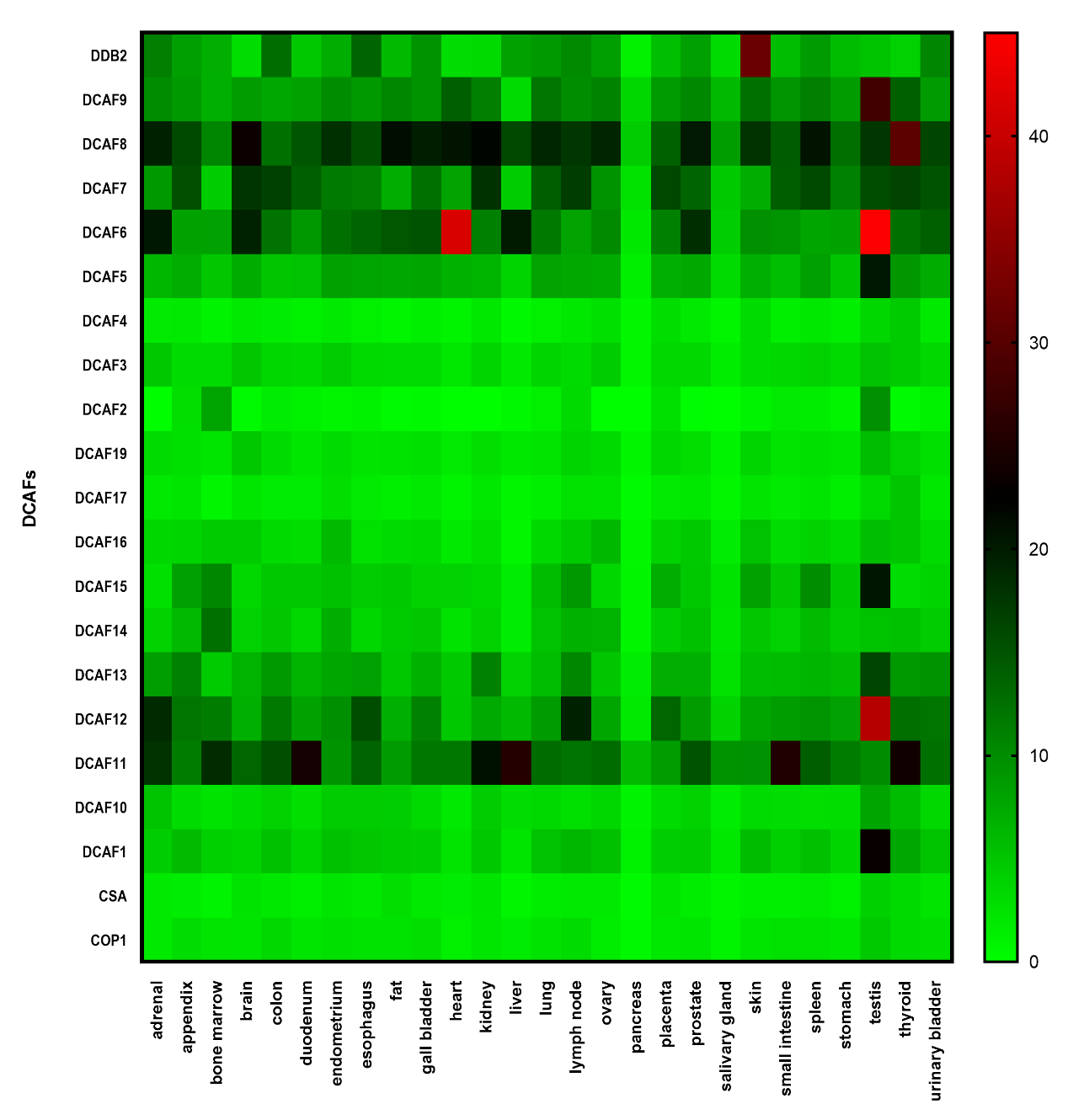


**Supplementary Figure 1**. **The expression profile of DCAFs in 27 human tissues**

Expression levels of different DCAFs in 27 human tissues were obtained from the NCBI website (https://www.ncbi.nlm.nih.gov/). Transcriptomic sequencing data of normal human tissues were analyzed, and a heatmap of DCAFs expression based on the RPKM values was created. Green indicates low expression levels, while red indicates high expression levels.


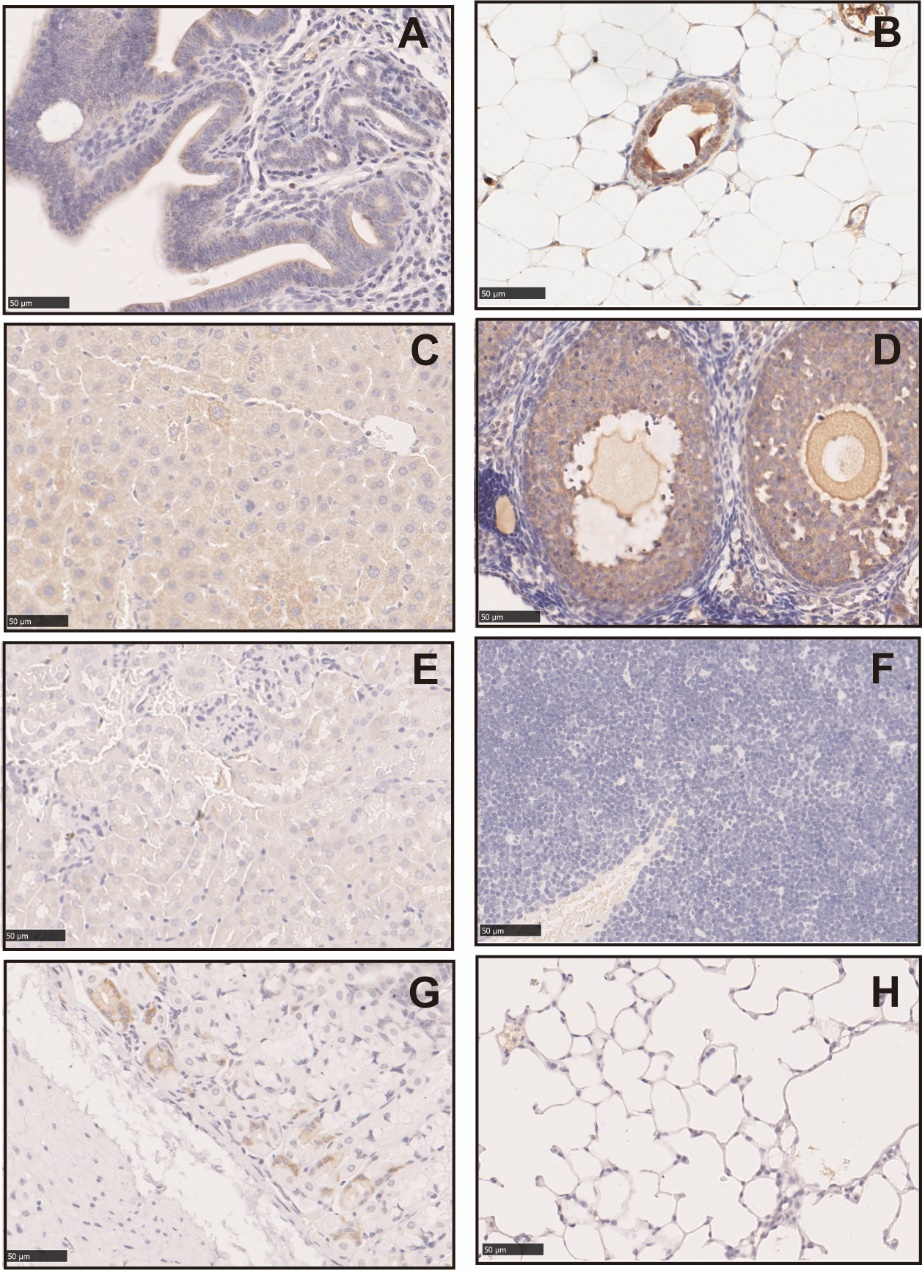


**Supplementary Figure 2. The expression profile of DCAF8 in mouse tissues**

Tissues from 12-week-old mice(n=3) as shown were paraffin-embedded, and sections were stained for DCAF8 by IHC. (A) Uterus; (B) Mammary gland; (C) Liver; (D) Ovaries; (E) Kidney; (F) Thymus; (G) Stomach; (H) Lung. Scale bar, 50 μm.


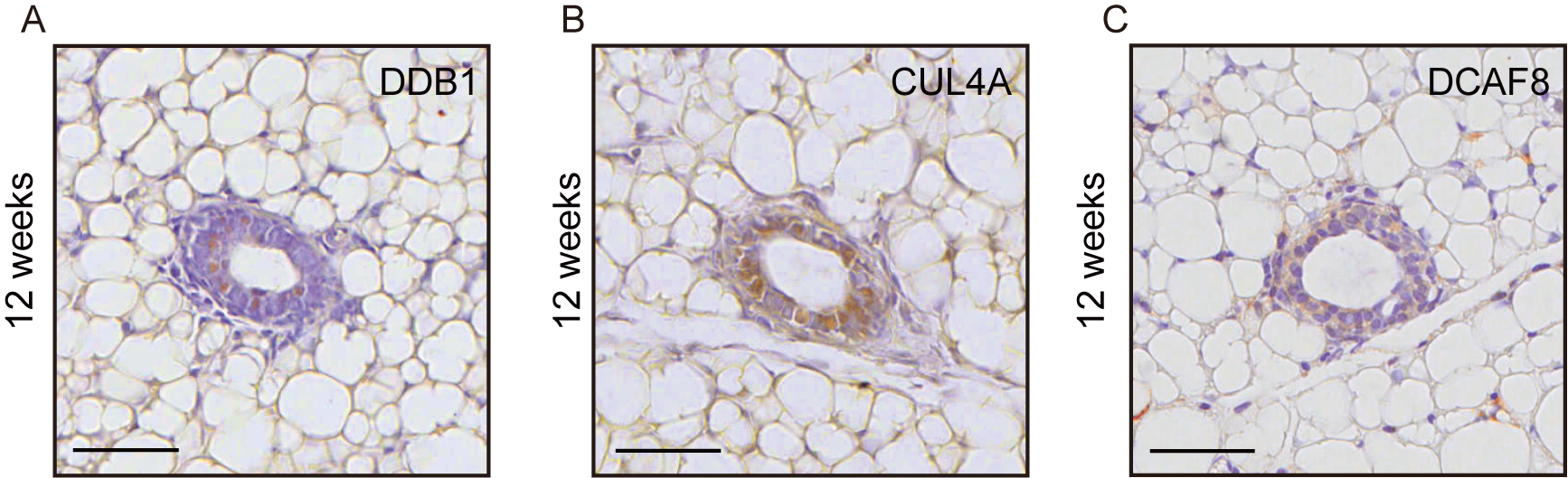


**Supplementary Figure 3. Immunohistochemistry staining of CRL4 in mouse mammary tissue**

Tissues from 12-week-old wild type mice as shown were paraffin-embedded, and sections were stained for DDB1 (A), CUL4A (B) and DCAF8 (C) by IHC. Scale bar, 50 μm.


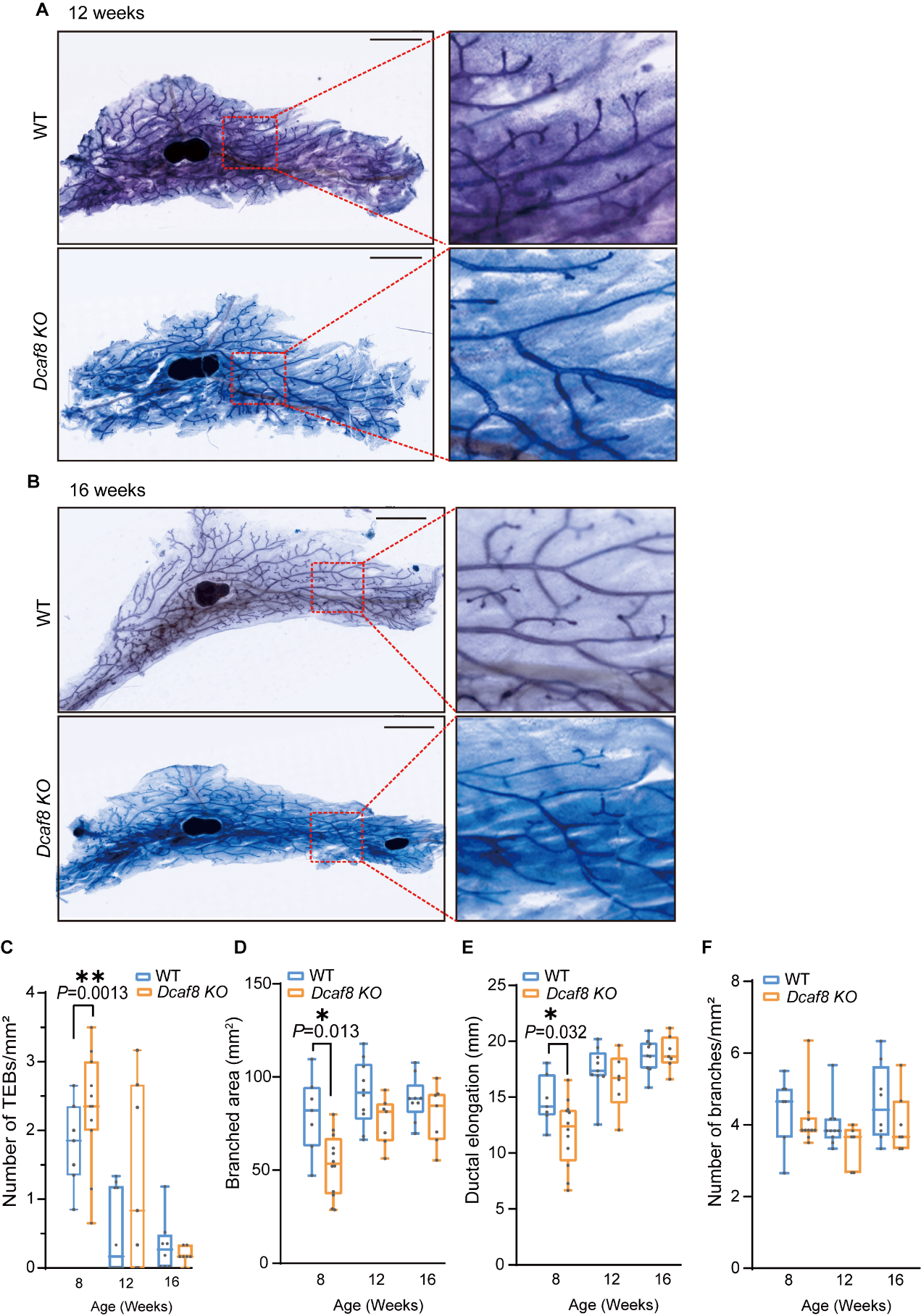


**Supplementary Figure 4. Quantitative analysis of the adult mouse mammary gland branching morphogenesis in *Dcaf8 KO* mice**

Quantitative analysis of mammary ducts in WT and *Dcaf8 KO* mice at 8 (WT, n=7; *Dcaf8 KO*, n=12), 12 (WT, n=10; *Dcaf8 KO*, n=7) and 16 weeks old (WT, n=9; *Dcaf8 KO*, n=7). Boxes: 25th and 75th percentiles, whiskers: minimum and maximum, central lines: medians; statistical analysis was performed using two-tailed *t*-tests. **P*<0.05.

(A-B) Whole mount toluidine blue staining of the mammary glands from WT and *Dcaf8 KO* mice at age of 12 and 16 weeks old, respectively. The right panel shows a magnified view of the local structures within the red frame on the left. Scale bar, 2.5 mm.

(C) The number of TEBs structures in the mammary ducts were determined as the average from three individual fields of view (FOV) per gland.

(D) The area of the mammary ductal tree was measured using ImageJ. The overall extension area of the mammary ducts (mm²) was calculated and plotted.

(E) The distance of ductal extension from the origin of the duct to the farthest edge (mm) was measured and plotted.

(F) The number of mammary duct branches, which was determined as the average of three individual fields of view (FOV) per gland was plotted.

**
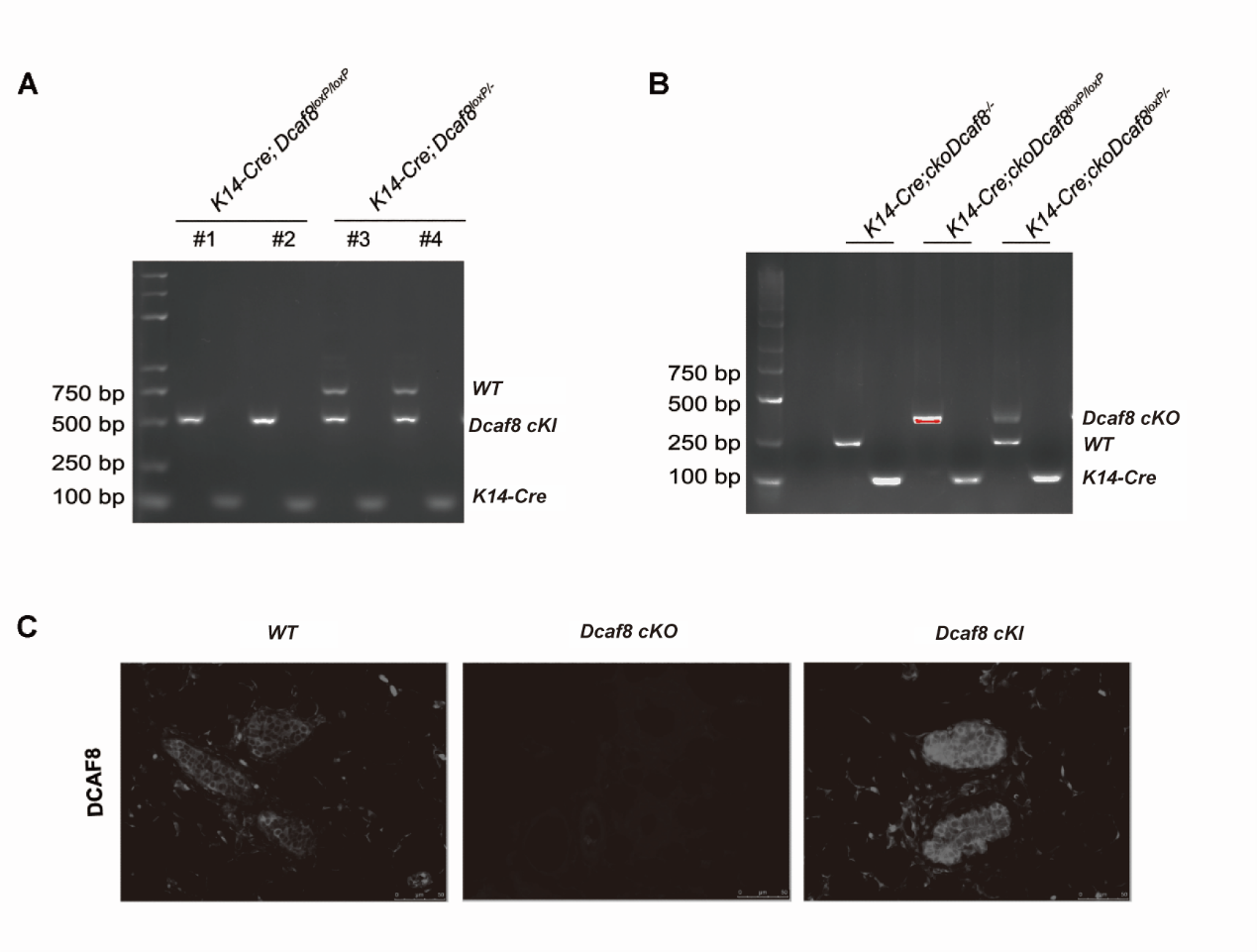
**

**Supplementary Figure 5. Confirmation of *Dcaf8* Conditional Knock-in (*Dcaf8 cKI*) and Knock-out (*Dcaf8 cKO*) Mouse Models**

1. Genotyping of *Dcaf8 cKI* mouse. Genomic DNAs were used for genotyping. The size of PCR product of *Dcaf8 KI* was 512 bp, while that of WT was 758 bp.
2. Genotyping of *Dcaf8 cKO* mouse. Genomic DNAs were used for genotyping. The size of PCR product of *Dcaf8 cKO* was 352 bp, while that of WT was 238 bp.
3. Representative images of DCAF8 immunofluorescence staining analysis of mammary gland tissue from 12-week-old *Dcaf8 cKO*, *Dcaf8 cKI* and WT mice. Scale bar, 50 μm.


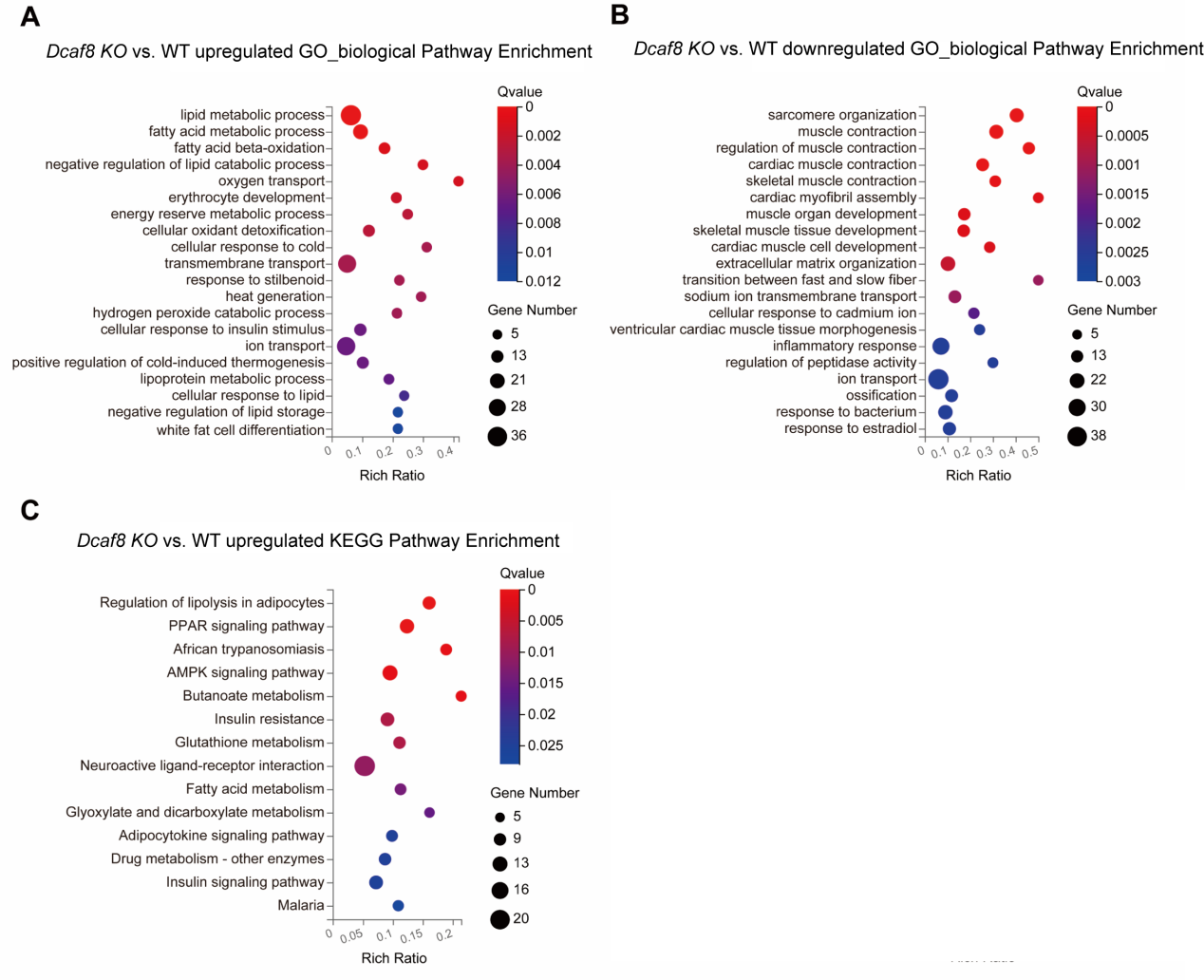


**Supplementary Figure 6. Functional enrichment analysis of differentially expressed genes between *Dcaf8* knockout mice and wild-type mice**

1. Upregulated GO biological pathway functional annotation of DEGs.
2. Downregulated GO biological pathway functional annotation of DEGs.
3. Upregulated KEGG pathway functional annotation of DEGs.
